# Prevalence and associated factors of diarrhea among under-five children in the Jawi district, Awi Zone Ethiopia, 2019. Community based comparative cross-sectional study

**DOI:** 10.3389/fped.2022.890304

**Published:** 2022-08-26

**Authors:** Dereje Nibret Gessesse, Abebe Aynalem Tarekegn

**Affiliations:** ^1^Department of Clinical Midwifery, School of Midwifery, College of Medicine and Health Sciences, Comprehensive Specialized Hospital, University of Gondar, Gondar, Ethiopia; ^2^Department of Human Anatomy, School of Medicine, College of Medicine and Health Sciences, Comprehensive Specialized Hospital, University of Gondar, Gondar, Ethiopia

**Keywords:** childhood diarrhea, model and non-model households, Jawi district, Ethiopia, under-five children

## Abstract

**Introduction:**

Although most deaths are preventable with simple and inexpensive measures, death from diarrhea accounts for one out of nine deaths in children worldwide which makes it the disease with the highest mortality rate in children under the age of five. Therefore, this study is aims to investigate diarrhea prevalence and risk factors among children under the age of five in Jawi district, Awi Zone, Ethiopia.

**Materials and methods:**

A comparative cross-sectional study was done among 440 study participants from March to June 2019. Data were collected with a face-to-face interviewer-administered questionnaire. Data was entered into EPI Info version 7 software and cleaned and analyzed using SPSS version 20 software. Binary logistic regression was done to assess independent factors associated with the dependent variable. A significant association was determined using an adjusted odds ratio at a confidence level of 95% and a *p*-value of less than or equal to 0.05.

**Results:**

In the current study, the overall under-five children diarrheal disease was found to be 15.5%. Diarrheal disease prevalence in model and non-model households was 10.9 and 20%, respectively. Shallow water [AOR: 6.12, 95%CI; (1.52, 24.58)], and maternal diarrhea [AOR: 4.11, 95%CI; (1.75, 9.61)] were determinants of childhood diarrhea. Place of birth [OR: 2.52, 95%CI (1.16, 5.49)] and maternal diarrhea [AOR: 3.50; 95%CI (1.28, 9.56)] in non-model households were also determinants of childhood diarrhea.

**Conclusion:**

Under-five children diarrheal disease was found to be high in the Jawi District. Thus, to decrease the disease prevalence in the study area, the health extension workers aim to better educate the mothers on how to handle diarrheal diseases. It is also better for concerned stakeholders to promote institutional delivery and to give access to safe water for the community.

## Background

According to World Health Organization (WHO), diarrhea is defined as “the passing of three or more loose or liquid stools per day or passing more frequently than is normal for the individual (1).” Although most deaths from diarrhea are preventable with simple, inexpensive measures, one in nine deaths in children worldwide is due to diarrhea. This means 2,195 children are dying per day due to diarrhea, which is equivalent to losing 32 school buses full of children each day or 801 thousand child deaths from diarrhea every year worldwide (2). According to a WHO report in 2015, diarrhea in children aged less than five causes 0.53 million deaths worldwide per year, which accounts for four in 1,000 live births (3) which makes it the most common cause of death worldwide (4). Another report surprisingly showed that child death is 15 times more in sub-Saharan Africa than in the developed world (5). Even though reports showed the decline of death due to childhood diarrhea in Ethiopia from 2000 to 2016, it still accounts for about 8% of deaths of under-five children and the number is still unacceptable (6). Some studies had been conducted in different parts of Ethiopia, but there were a large gap in the burden of disease and determinants between studies and showed a range of 11–31% (7–12). The recent findings suggested that interventions such as pure water consumption, good hygiene and sanitation, breastfeeding, complementary feeding, zinc and vitamin A supplementation, and Rota vaccination were effective to prevent diarrhea in children under five (13). Despite these suggestions, the burden of disease in children is high, and community-based studies are limited, especially in the proposed district. Even these limited studies had great differences in disease burden and factors related to child diarrhea.

Therefore, this study aimed to assess the prevalence and determinants of diarrheal diseases in children under 5 years of age residing in modeled households and non-modeled HHs in the Jawi district, Amhara regional state. Thus, the results of the study will be used to develop prevention strategies at the community level in the district. The findings of this study will also be used to put priority settings on the prevention of under-five diarrheal diseases in the community level.

## Materials and methods

**FIGURE 1:**
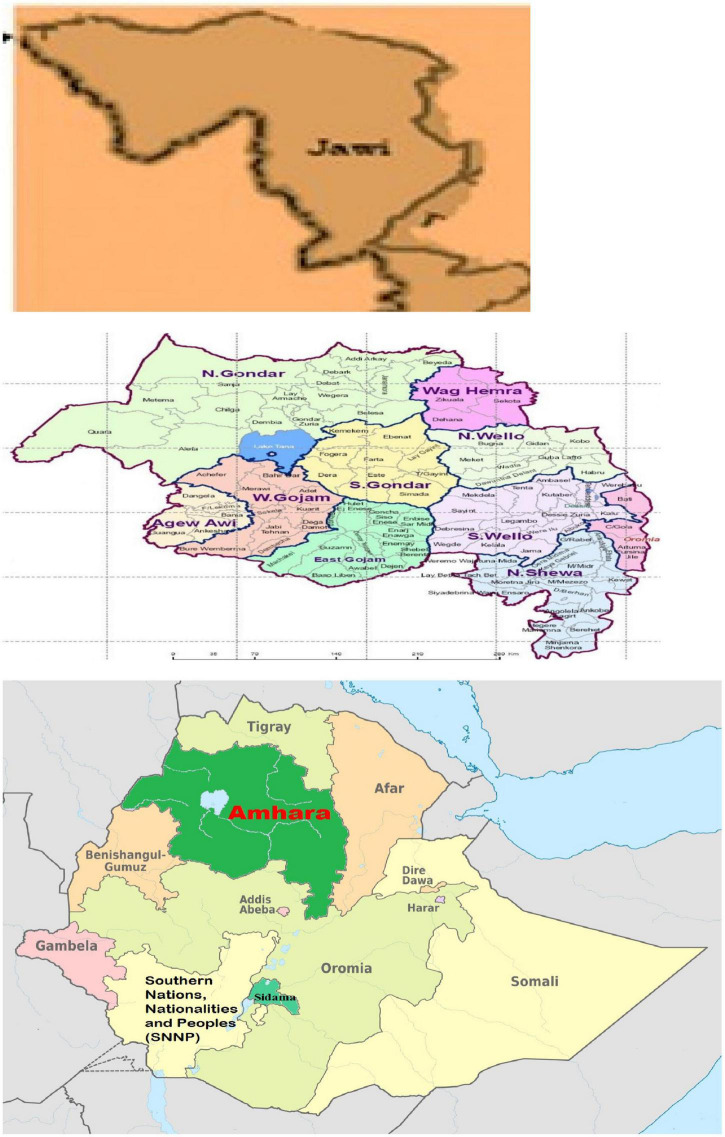
Map showing Jawi District, Awi Zone, Ethiopia.

A community-based comparative cross-sectional study was conducted from May to June 2019. The Jawi district is found in the Western Amhara region 540 km away from Addis Ababa, the capital of Ethiopia, and 200 km away from Bahir Dar, the capital city of Amhara regional state. A total of 163,102 people live in this district, of which 50% are female. From the total population, about 19,399 are under-five children. There are about 35 governmental health institutions (28 health posts, six health centers, and one primary hospital) and 38 non-governmental health institutions (four medium clinics and 34 primary clinics) in the district. The total health workforces were about 215 of which 63 were found to be health extensions in the district. The water source of the district was mainly shallow (425), followed by a hand pump (100) and a water pipe in three kebeles (Figure 1). All under-five children were included, but any children whose caregivers were mentally ill and had a hearing problem were excluded from the study.

The sample size was determined by using a double population proportion formula considering, 6.4% the proportion of diarrhea in extension modeled HHs and 25.5% the proportion of diarrhea in extension none modeled HHs among under-five children (11), 95% confidence level and 80% power.

In the study that was conducted in the Sheko district the n1 was 275 and n2 was 550 with a ratio of two, based on this information I was calculated and pulled *p* and *q* values. The pulled *p*-value is 0.1913 and *q* is 0.809.


$$n_{1}=n_{2}=\frac{{\left[Z\alpha /2\;2p^{-}q^{-}+z1-\beta \left(\left(p1q1\right)+\left(p2q2\right)\right)\right]}^{2}}{\left(P1-p2\right)}$$

$$n_{1}=n_{2}=\frac{\begin{array}{l} {}[1.962\left(0.1913\right)\left(0.809\right)+0.842((6.4)\left(25.5\right)\\ \;\;\;\;\;\;+(0.936)(0.745){]}^{2} \end{array}}{{\left(0.064-0.255\right)}^{2}}=100$$

Therefore, by considering the design effect of 2 and 10 percent non-response rates, the total sample size was 440.

n1 = number of exposed (number of model households).

n2 = number of non-exposed (number of non-model households).

p1 = proportion of diarrheal case among children living model households.

p2 = proportion of diarrheal case among children living in non-model households ^–^p = pulled *p*-value; ^–^q = pulled *q* value.

The largest sample size obtained using the associated factor for this study was 148. By considering 10% of non-response and two design effects, the largest sample size was estimated to be 326 which was less than the sample size calculated above. Therefore, the final sample size for this study was 440.

In the study district, there were two health extension modeled and 26 non-modeled kebeles. The kebeles were stratified to health extension modeled and not modeled. From the non-model kebeles, three kebeles were randomly selected using the lottery method and two kebeles from the modeled stratum were surveyed. From each selected kebele, households that have at least one child were enumerated by data collectors. Then, systematic random sampling was used to select households. The lottery method was used if there was more than one child in the household. When a household was closed during data collection, the next household was included.

Diarrhea was defined as passing three or more loose or watery stools in 24 h in the household within the 2 weeks before the survey, as reported by the mother/caretaker of the child (14). Model household was defined as having a household head/caregiver who had taken basic training for 96 h and graduated on the 16 health extension packages (15). Non-model family was defined as having a household head/caregiver who had not taken basic training on the 16 health extension packages (15). Handwashing at a critical time was based on whether a mother/caregiver practiced all simple hand washings before food preparation, before child feeding, after child cleaning, and after latrine visiting. If these criteria were met, it was considered to be “all practiced” otherwise it was considered to be “partially practiced (16).” Proper refuse disposal was defined as a way of disposal of refuse which includes burning, buried in a pit or stored in a container, composting, and disposed of at a designated site, whereas disposal in an open field was considered as improper refuse disposal (14).

A pretested, face-to-face interviewer-administered structured questionnaire was used to collect the desired sample size. The tool was first developed in English and translated to the local language then back to English to keep the tool consistent. The data was collected by four nurses and there was one health officer for supervision. Data was collected from the mother, father, or other caregivers using an interviewer-administered questionnaire. Data collector nurses and a supervisor were trained on basic interviewing techniques. The data were checked daily for its completeness by supervisors and principal investigators. The tool was pre-tested in 5% of the sample in deke 01 kebele. Data was entered into EPI Info version 7 software and cleaned and analyzed using SPSS version 20 software. Frequency, percentage, and mean were used to describe the characteristics of study participants. Binary logistic regression was carried out to assess independent factors associated with the dependent variable. A significant association was determined using an adjusted odds ratio at a confidence level of 95% and a *p*-value of less than or equal to 0.05.

## Results

### Socio-demographic characteristics of study participants

In the current study, a total of 440 study participants were included making the response rate 100%. Among the total study participants, half of them were from model households. The mean age of the study participants was 27.7 (±14.8 SD) months in model households and 33.6 (±13.9 SD) months in non-model households. Of the total study participants, the mean height was 84.6 (±11.4 SD) centimeters in model and 82.3 (±12.8 SD) centimeters in non-model HHs (Table 1).

**TABLE 1:** Socio demographic characteristics of respondents in Jawi district, Awi Zone, southwest Ethiopia, 2019.

Variables	Model HHs		Non-model HHs	
		
	Diarrhea			

	Yes (%)	No (%)	Yes (%)	No (%)
**Sex of child**				
Male	10 (41.7)	92 (46.9)	21 (47.7)	85 (48.3)
Female	14 (58.3)	103 (52.6)	23 (52.3)	91 (51.7)
**Place of birth**				
Home	3 (12.5)	35 (17.9)	13 (29.5)	26 (14.8)
Institution	21 (87.5)	161 (82.1)	31 (70.5)	150 (85.2)
**Ethnicity**				
Amhara	17 (70.8)	151 (77.0)	21 (47.7)	95 (54.0)
Awi	7 (29.2)	45 (23.0)	23 (52.3)	81 (46.0)
**Religion**				
Orthodox	16 (66.7)	159 (81.1)	43 (97.7)	174 (98.9)
Muslim	8 (33.3)	37 (18.9)	1 (2.3)	2 (1.1)
**Maternal education status**				
No education	10 (41.7)	74 (37.8)	30 (68.2)	131 (74.4)
Primary	11 (45.8)	99 (50.5)	14 (31.8)	44 (25.0)
Secondary & above	3 (12.5)	23 (11.7)	0 (0.0)	1 (0.6)
**Maternal occupation**				
House wife	17 (70.8)	134 (68.4)	9 (20.5)	12 (6.8)
Farmer	3 (12.5)	25 (12.8)	33 (75.0)	160 (90.9)
Government employed	2 (8.3)	12 (6.1)	0 (0.0)	1 (0.6)
Non-governmental organization	2 (8.3)	15 (7.7)	1 (2.3)	3 (1.7)
Not employed	0 (0.0)	10 (5.1)	1 (2.3)	0 (0.0)
**Father’s occupation**				
Government employed	8 (33.3)	19 (9.7)	0 (0.0)	2 (1.1)
Non-governmental organization	7 (29.2)	94 (48.0)	4 (9.1)	4 (2.3)
Farmer	8 (33.3)	71 (36.2)	39 (88.6)	168 (95.5)
Not employed	1 (4.2)	12 (6.1)	1 (2.3)	2 (1.1)
**Father’s education status**				
No education	7 (29.2)	36 (18.4)	20 (45.5)	99 (56.3)
Primary	9 (37.5)	122 (62.2)	24 (54.5)	75 (42.6)
Secondary & above	8 (33.3)	38 (19.4)	0 (0.0)	2 (1.1)
**Age of caregiver**				
15–24	8 (33.3)	52 (26.5)	4 (9.1)	24 (13.6)
25–34	10 (41.7)	111 (56.6)	24 (54.5)	99 (56.3)
35–49	6 (25.0)	33 (16.8)	16 (36.4)	53 (30.1)
**Number of under 5 children/HHs**				
One	14 (58.3)	138 (70.4)	32 (72.7)	138 (78.4)
Two	10 (41.7)	57 (29.1)	11 (25.0)	37 (21.0)
Three	0 (0.0)	1 (0.5)	1 (2.3)	1 (0.6)
**Family size**				
Less than or equal to five	19 (79.2)	155 (79.1)	24 (54.5)	97 (55.1)
Greater than five	5 (20.8)	41 (20.9)	20 (45.5)	79 (44.9)

HHs, households.

### Environmental and personal factors

All study households had a latrine, of which 167 (75.9%) of model HHs and 5 (2.3%) of non-model HHs used an improved type of latrine. Of these, 193 (87.7%) and 82 (37.3%) latrines were clean among model and non-model households respectively. 95 (44.5%) model households had a hand washing facility on their latrine but only 5 (2.3%) non-model households had a hand washing facility. Among 220 model and 220 non-model households, 210 (95.5%) and 165 (75%) households used separate kitchens, respectively. The median distance to fetch water for model households was 6 m with a minimum distance of 1 m and a maximum distance of 100 m away from their home, and for non-model households the median was 10 m with a minimum distance of 2 m and a maximum distance of 100 m away from their home. The mean distance of the latrine from the HHs was 14.3 (±7.8SD) meters from which the mean distance away from the model and non-model HHs was 12 (±5.38SD) meters and 16.6 (±9.13SD) meters respectively (Table 2).

**TABLE 2:** Environmental and personal factors, and maternal and child health condition of respondents in Jawi district, Awi Zone, southwest Ethiopia, 2019.

Variables	Model HHs		Non-model HHs	
		
	Diarrhea			

	Yes (%)	No (%)	Yes (%)	No (%)
**Type of latrine**				
Improved	17 (70.8)	150 (76.5)	1 (2.3)	4 (2.3)
Unimproved	7 (29.2)	46 (23.5)	43 (97.7)	172 (97.7)
**Clean latrine**				
Yes	22 (91.7)	171 (87.2)	21 (47.7)	61 (34.7)
No	2 (8.3)	25 (12.8)	23 (52.3)	115 (65.3)
**Latrine has hand washing**				
Yes	9 (37.5)	89 (45.4)	1 (2.3)	4 (2.3)
No	15 (62.5)	107 (54.6)	43 (97.7)	172 (97.7)
**Waste disposal pit**				
Yes	17 (70.8)	102 (52.0)	1 (2.3)	2 (1.1)
No	7 (29.2)	94 (48.0)	43 (97.7)	174 (99.9)
**Domestic animal live in house**				
Yes	1 (4.2)	6 (3.1)	1 (2.3)	1 (0.6)
No	23 (95.8)	190 (96.9)	43 (97.7)	175 (99.4)
**Water source for domestic use**				
Pipe	9 (37.5)	126 (64.3)	1 (2.3)	1 (0.6)
Water pump	9 (37.5)	31 (15.8)	0 (0.0)	1 (0.6)
Shallow	6 (25.0)	39 (19.9)	43 (97.7)	174 (98.9)
**Maternal diarrhea**				
Yes	3 (12.5)	6 (3.1)	8 (18.2)	11 (6.3)
No	21 (87.5)	190 (96.9)	36 (81.8)	165 (93.7)
**Albenedazole**				
Yes	7 (29.2)	58 (29.8)	31 (70.5)	100 (56.8)
No	6 (25.0)	64 (32.7)	5 (11.4)	42 (23.9)
Age < 2 years	11 (45.8)	74 (37.8)	8 (18.2)	34 (19.3)
**Rota vaccine taken**				
Yes	24 (100)	189 (96.4)	42 (95.5)	170 (96.6)
No	0 (0.0)	4 (2.0)	1 (2.3)	4 (2.3)
Age < 6 weeks	0 (0.0)	3 (1.5)	1 (2.3)	2 (1.1)
**Measles vaccine taken**				
Yes	17 (70.8)	171 (87.2)	40 (90.9)	163 (92.6)
No	2 (8.3)	7 (3.6)	2 (4.5)	3 (1.7)
Age < 9 month	5 (20.8)	18 (9.2)	2 (4.5)	10 (5.7)

HHs: households.

### Maternal and child health condition

Maternal diarrheal prevalence was 6.4% (95%CI, 4.1, 8.6), of which the prevalence based on the model type was 4.1% in model and 8.6% in non-model HHs. Among the study participants, 313 were greater than or equal to 2 years during the study period among them 196 (62.6%) were dewormed with albendazole. Of which 65 (33.2%) were from model households and 131 (66.8%) were from non-model households. 405 (92.0%) of the children were above the age of 9 months, of which 391 (96.5%) were vaccinated against measles and among them, 188 (85.5%) were from model households and 203 (92.3%) were from non-model households. 434 (98.6%) of the study participants were older than 6 weeks, and among them 425 (97.9%) were vaccinated against Rota, of which half of the children were in model and non-model HHs (Table 2).

### Knowledge and behavioral factors of caregivers

Of the total caregivers, 272 (61.96%) of them know at least one mode of transmission of diarrhea, of which 160 (58.8%) of them were residing in model HHs and 112 (41.2%) were residing in non-model HHs. The caregivers were asked when they started supplementary food for their children 423 (96.1%) answers were at or after the age of 6 months, of which 407 (96.2%), 3 (0.7%), and 12 (2.8%) answers at 6, 8, and 12 months, respectively (Table 3).

**TABLE 3:** Knowledge and behavioral factors of respondents in Jawi district, Awi Zone, southwest Ethiopia, 2019.

Variables	Model HHs		Non-model HHs	
		
	Diarrhea			

	Yes (%)	No (%)	Yes (%)	No (%)
**Mode of transmission**				
Yes	19 (79.2)	141 (71.9)	22 (50.0)	90 (51.1)
No	5 (20.8)	55 (28.1)	22 (50.0)	86 (48.9)
**Method of prevention**				
Yes	18 (75.0)	133 (67.9)	14 (31.8)	75 (42.6)
No	6 (25.0)	63 (32.1)	30 (68.2)	101 (57.4)
**Homemade treatment**				
Yes	6 (25.0)	70 (35.7)	3 (6.8)	16 (9.1)
No	18 (75.0)	125 (63.8)	41 (93.2)	160 (90.9)
**Critical time for hand washing**				
Yes	21 (87.5)	156 (79.6)	40 (90.9)	168 (95.5)
No	3 (12.5)	40 (20.4)	4 (9.1)	8 (4.5)
**Supplementation**				
At ≥ 6 month	21 (87.5)	183 (93.4)	43 (97.7)	176 (100)
At < 6 month	3 (12.5)	13 (6.6)	1 (2.3)	0 (0.0)

HHs, households.

### Prevalence of diarrhea

Diarrheal disease prevalence of under-five children in the study district in the 2 weeks prior to the interview was 15.5% (95% CI, 12.5–18%). The occurrence of diarrheal disease morbidity in model and non-model HHs was 10.9% (95% CI; 6.8–15.0%) and 20% (95%CI; 15.0–25.5%), respectively.

### Independent factors associated with childhood diarrhea

To avoid excess numbers and unstable estimates, variables were screened by bivariable logistic regression, and variables with a *p*-value ≤ 0.25 were included in multivariable logistic regression analysis. Accordingly, the odds of child diarrhea from HHs who use shallow water were 6.12 times more likely compared to those who use water pump as water source [AOR: 6.12, 95%CI; (1.52, 24.58)]. The odds of child diarrhea were 12.8 times more likely in the Orthodox Christian religion compared to the Muslim religion [AOR: 12.8, 95%CI; (3.3, 50)]. The odds of child diarrhea were 4.1 times more common in HHs in which the mother had diarrhea [AOR: 4.11, 95%CI; (1.75, 9.61)] as shown in Table 4.

**TABLE 4:** Independent factors associated with childhood diarrhea of respondents in Jawi district, Awi Zone, southwest Ethiopia, 2019.

Variables	Child diarrhea		COR	AOR
			
	Yes	No		
**Maternal diarrhea**				
Yes	11	17	4.03 (1.80,9.04)	4.11 (1.75,9.61)**
No	57	365	1	1
**Source of water**				
Shallow	49	213	1.22 (0.55, 1.43)	6.12 (1.52,24.58)*
Pipe	10	127	0.34 (0.17, 0.70)	0.526 (0.15, 1.83)
Water pump	9	32	1	1
**Religion**				
Orthodox	59	333	1.3 (0.6, 2.8)	12.8 (3.3, 50)*
Muslim	9	9	1	1
**Household type**				
Model	24	196	1	1
Non-model	44	176	2.04 (1.19,3.49)	1.54 (0.50, 4.79)
**Type of latrine**				
Improved	18	154	1	1
Unimproved	50	218	1.96 (1.10,3.49)	1.13 (0.40, 3.19)
**Ethnicity**				
Awi	30	126	1.54 (0.91,2.61)	1.18 (0.66, 2.11)
Amhara	38	248	1	1
**Place of birth**				
Home	16	61	1.57 (0.84, 2.93)	1.72 (0.88, 3.35)
Institution	52	311	1	1
**Hand washing facility**				
Yes	10	93	1	1
No	58	279	1.93 (0.95, 3.94)	1.30 (0.52, 3.25)
**Mebendazole supplementation**				
Yes	38	158	2.32 (1.13, 4.73)	2.13 (0.90, 4.54)
No	11	106	1	1

*Significance at *p*-value ≤ 0.05, **significance at *p*-value ≤ 0.001; COR, crude odds ratio; AOR, adjusted odds ratio. The bold letter-significant association at *P*-value < 0.05.

### Determinants of childhood diarrhea among model and non-model households

After stratifying study subjects into model and non-model HHs, bivariate screening was done and variables with a *p*-value ≤ 0.25 were included in multivariable logistic regression. No variable had an association with childhood diarrhea in model HHs. On the other hand, among non-model households, the risk of getting diarrhea in children who delivered at home was 2.5 times higher compared to children who delivered at the institution [OR: 2.52, 95%CI (1.16, 5.49)]. The odds of child diarrhea were 3.5 times more likely among children whose mother had diarrhea as compared to those whose mother did not have diarrhea in non-model HHs [OR: 3.50; 95%CI (1.28, 9.56)] as shown in Table 5.

**TABLE 5:** Independent factors associated with childhood diarrhea among non-model households (HHs) of respondents in Jawi district, Awi Zone, southwest Ethiopia, 2019.

Variables	Child diarrhea		COR	AOR
			
	Yes	No		
**Maternal diarrhea**				
Yes	8	11	3.33 (1.25, 8.88)	**3.50 (1.28, 9.56)**
No	36	165	1	1
**Place of birth**				
Home (33.3) (66.7)	13	26	2.42 (1.12, 5.23)	**2.52 (1.16, 5.49)**
Institution	31	150	1	1
**Clean latrine**				
Yes	23	115	1	1
No	21	61	1.72 (0.88, 3.35)	1.61 (0.8, 3.23)
**Mebendazole supplementation**				
Yes	31	100	2.60 (0.95, 7.14)	2.70 (0.95, 7.69)
No	5	42	1	1

COR, crude odds ratio; AOR, adjusted odds ratio. The bold letter-significant association at *P*-value < 0.05.

## Discussion

In the current study, the overall prevalence of under-five children diarrheal disease was found to be 15.5%. The result was relatively lower than the study done in Arba Minch district (31%) (17). The prevalence of diarrheal disease is also lower compared to other studies conducted in Ethiopia: 19.6% in Shebedino District (18), 21.5% in Jabithennan (19), 22.0% in Ethiopia (20), 22.1% in Benishangul Gugmuz (21), 22.5% in Eastern Ethiopia (10), 26% in Mbour, Senegal (22), 26.1% in Nomadic (23), 27% in Jigjiga district (24), and 30.5% in Arba Minch (17). The variation may be related to the differences in caregivers’ basic environmental and behavioral characteristics. However, the result was high compared to the study conducted in Addis Ababa (11.9%) (25).

A possible explanation for the discrepancy is the difference in socioeconomic development, there could be better awareness about diarrhea prevention mechanisms which could decrease the disease prevalence, and this showed that more attention needs to be paid to the reduction of child morbidity in the area to reduce child death.

The diarrheal morbidity of children under 5 years of age among model and non-model HHs was about 11 and 20% respectively. This figure was higher than a comparative study done in Hawassa (9%) and (14%) among model and non-model HHs (11). This might be because the current study includes residences from both urban and rural areas. The methods of health education and health service delivery between rural and urban residents are possibly different. Urban residents might have better information on how to handle waste products and how to care for their child by considering prevention mechanisms of diarrhea.

However, this study had a higher prevalence among model HHs and a lower prevalence among non-model HHs compared to a study done in Sheko district, southwest Ethiopia (6.4% and 25.5%, respectively) (16). This difference could be due to different socio-demographic characteristics of the mothers and differences in weather conditions.

The source of water used for household utility had a significant impact on diarrheal diseases. This study showed that using shallow water is a risk factor for developing diarrhea compared to using water from a water pump. This is because shallow water is unprotected from contamination with animal and human waste, which may contain a range of pathogenic microorganisms causing an increased risk of a range of diarrheal diseases. This is in line with other studies (23, 24).

Maternal diarrhea was another variable that was associated with childhood diarrhea morbidity. Children whose mothers had diarrheal diseases had a high chance of developing diarrhea. This is because mothers with a disease contaminate the food, water, and utensils and directly when handling their children. This is consistent with other study findings (11).

In the study, the place of birth of their index child was significantly associated with childhood diarrhea among non-model households. Home delivery of their index child increased the likelihood of developing a diarrheal disease compared to delivery at an institution. This can be explained by the fact that mothers who give birth at an institution can have better information on how to care for their children and on how to prevent diarrheal disease. Moreover, mothers who gave birth in an institution are given information on exclusive breast feeding, which might decrease the chance of getting an infection that may lead to diarrheal disease. This was not associated significantly in the study done in Shebedino District (18) and in other studies.

### Limitation

Caregivers of the child were asked about their children’s health status in the 2 weeks before the survey which might measure their perceptions rather than actual morbidity that might affect the result. Perceptions of illness may not be the same in different caregivers.

## Conclusion

Childhood diarrhea was found to be prevalent in the study District. The factors that were associated with childhood diarrhea were maternal diarrhea, the source of water for domestic use, and religion. Factors associated with childhood diarrhea among non-model HHs were the place of birth for their index child and maternal diarrhea.

Thus, to decrease childhood diarrhea in the study area, the health extensions should better educate the community on how to dispose of solid and liquid wastes, the pit they are going to use for waste disposal, and how mothers can handle the contraction of a diarrheal disease. for concerned stakeholders, it is also better to promote institutional delivery and to give access to the community for safe water.

## Data availability statement

The raw data supporting the conclusions of this article will be made available by the authors, without undue reservation.

## Ethics statement

The studies involving human participants were reviewed and approved by the University of Gondar internal review board with a reference number of V/P/RCS/05/2710/2019. Written informed consent was obtained from the study participants before commencing the data collection.

## Author contributions

Both authors contributed to the conception of the study to final approval of the version to be published.

## Abbreviations

HHs: households
WHO: World Health Organization
AOR: adjusted odds ratio
CI: confidence interval
COR: crude odds
SD: standard deviation
SPSS: statistical package for social science.
